# An EEG-Based Person Authentication System with Open-Set Capability Combining Eye Blinking Signals

**DOI:** 10.3390/s18020335

**Published:** 2018-01-24

**Authors:** Qunjian Wu, Ying Zeng, Chi Zhang, Li Tong, Bin Yan

**Affiliations:** 1 China National Digital Switching System Engineering and Technological Research Center, Zhengzhou 450001, China; qunjian1992@163.com (Q.W.); yingzeng@uestc.edu.cn (Y.Z.); zcboluo@hotmail.com (C.Z.); tttocean@163.com (L.T.); 2 Key Laboratory for NeuroInformation of Ministry of Education, School of Life Science and Technology, University of Electronic Science and Technology of China, Chengdu 610000, China

**Keywords:** person authentication, EEG, eye blinking, multi-task, score fusion, open-set authentication, permanence capability

## Abstract

The electroencephalogram (EEG) signal represents a subject’s specific brain activity patterns and is considered as an ideal biometric given its superior forgery prevention. However, the accuracy and stability of the current EEG-based person authentication systems are still unsatisfactory in practical application. In this paper, a multi-task EEG-based person authentication system combining eye blinking is proposed, which can achieve high precision and robustness. Firstly, we design a novel EEG-based biometric evoked paradigm using self- or non-self-face rapid serial visual presentation (RSVP). The designed paradigm could obtain a distinct and stable biometric trait from EEG with a lower time cost. Secondly, the event-related potential (ERP) features and morphological features are extracted from EEG signals and eye blinking signals, respectively. Thirdly, convolutional neural network and back propagation neural network are severally designed to gain the score estimation of EEG features and eye blinking features. Finally, a score fusion technology based on least square method is proposed to get the final estimation score. The performance of multi-task authentication system is improved significantly compared to the system using EEG only, with an increasing average accuracy from 92.4% to 97.6%. Moreover, open-set authentication tests for additional imposters and permanence tests for users are conducted to simulate the practical scenarios, which have never been employed in previous EEG-based person authentication systems. A mean false accepted rate (FAR) of 3.90% and a mean false rejected rate (FRR) of 3.87% are accomplished in open-set authentication tests and permanence tests, respectively, which illustrate the open-set authentication and permanence capability of our systems.

## 1. Introduction

With the development of non-invasive brain-computer interfaces (BCIs), electroencephalograms (EEGs) have become a hotspot in many research fields because of their high time resolution, portability and relatively low cost [1]. One of the research topics is the use of EEGs as a biometric trait.

Traditional biometric traits, such as faces [2], fingerprints [3], voiceprints [4], and irises [5], have a high degree of discrimination and are widely used. However, most of these traits are easy to steal and forge given their exposure to the external world. For example, face features can be extracted from the user’s photos and fingerprints can be easily forged from the things that the user touches. EEG is the electrical recording of brain activity, represented as voltage fluctuations resulting from ionic current flows within the neurons of the brain [6]. EEGs can be a novel biometric trait because an individual’s neural activity pattern is unique [7] and imitating one’s mind is impossible [8]. This trait can change the traditional notion of “pass-word” into the “pass-thought”. Furthermore, external pressure will significantly influence EEG signals. Thus, if a user is forced to enter the password, his high stress could be detected, stopping the access. This property is referred to as ‘‘circumvention’’ within the biometry field [9].

Numerous EEG-based identity authentication methods have been proposed based on unique EEG features. These methods can be roughly divided into two categories of spontaneous or evoked EEGs based on the absence or presence of a stimulus. The former includes rest eyes-open/eyes closed (REO/REC), whereas the latter involves visual evoked potentials (VEPs), mental tasks, and emotional stimuli.

In 1999, Poulos et al. developed the first identity authentication system based on EEG signals [10]. They collected the EEG data of four users and 75 imposters under REC conditions. Auto- regressive parameters and a learning vector quantization network were adopted, and the correct recognition rates of 72% to 84% were achieved. Palaniappan et al. constructed a dataset of VEP signals from 20 subjects [11]. The subjects focused on recognizing stimulus images from the Snodgrass and Vanderwart picture set [12]. The highest accuracy of 92.84% was obtained using the simplified fuzzy adaptive resonance theory. Sun et al. collected the EEG signals of nine subjects while they imagined moving their right or left index finger. The researchers concluded that imagining the movements of the left index finger is more appropriate for identity identification with an accuracy of 95.6% [13]. Yeom et al. [14] used self- or non-self-face images as stimulus to evoke subject-specific brain activities based on neurophysiological evidence from both EEG [15] and fMRI [16]. A unique subject-specific brain-wave pattern called visual self-representation was elicited by their experimental paradigm. They obtained an average accuracy of 86.1% across 10 subjects using non-liner support-vector machine. Sharma et al. [17] proposed an individual identification method through motor movement and motor imagination. Using wavelet transform and neural network classifier, they found that performance based on motor imagination is better than motor movement. Although these previous works obtained successful performances, the internal uniqueness of the elicited EEG signals remains unconfirmed. Moreover, most of the EEG-based authentication methods are under off-line analysis or require too much time for one-time authentication. Thus, evoking a stronger and more stable individual difference in less time is crucial in EEG-based authentication systems.

In this paper, we firstly propose a novel experimental paradigm using self- or non-self-face images that are organized by rapid serial visual presentation (RSVP) [18]. In the RSVP paradigm, the stimulus images are presented one-by-one in a certain order and in the same position of the screen for the same presentation time. The RSVP paradigm can present a large number of stimuli in a short time and thus elicit strong event-related potentials (ERPs) [19]. The latency, amplitude, or shape of ERPs vary across subjects because of the inherent subject-to-subject variation in the neural pathways of the brain [20]. Compared with previous works, we elicited stronger subject-specific ERPs in less time through our face RSVP paradigm. Thus, the real-time capability and accuracy of the system are significantly improved. A preliminary partial version of our research was proposed in [21].

Introducing new biometric features is a common way to enhance the identity authentication systems. For instance, Pham et al. [22] fused user’s rich information (such as age and gender) into EEG features and got a much lower error rate. Patel et al. [23] combined the hand synergies features with EEG features together and obtained a more stable and precise performance. However, the above methods did not fuse the EEG signal with bioelectric signals. Thus, there are still some security risks. An exciting research was completed by Abo-Zahhad et al. They find that the eye blinking electrooculogram (EOG) are diverse from different person and can be a novel biometric [24]. Moreover, they proposed a multi-level approach based on the fusion of EEG and eye blinking signals, and a lower error rate was obtained [25]. The eye blinking mainly affects the EEG signal in the frontal scalp regions. Thus, in regular EEG acquisition, the eye blinking signals are regarded as artifacts and usually removed from raw EEG signals using independent component analysis (ICA) [26]. However, when regarding eye blinking signals as biometrics, the signals collected from the frontal electrodes are crucial, especially in FP1 and FP2.

In this paper, inspired by Abo-Zahhad et al., we employ the eye blinking signals to enhance our EEG-based person authentication system. Compared to the previous work, we have modified the feature extraction and classification methods. And for the multi-task authentication system, a new score level fusion of EEG and eye blinking signals approach based on least square method is proposed. Finally, in order to improve the system’s robustness, we recruit 15 imposters for each user. In this way, richer feature information are added to enhance the classifier’s performance, so that the authentication system is more stable. Moreover, open-set authentication tests for additional imposters and permanence tests for users are conducted to simulate the practical scenarios, which have never been employed in previous EEG-based person authentication systems. The experiment results illustrate the practicability of proposed multi-task authentication system.

## 2. Materials and Methods

### 2.1. Main Framework Design

#### 2.1.1. Main Framework of the Authentication System

**Figure 1:**
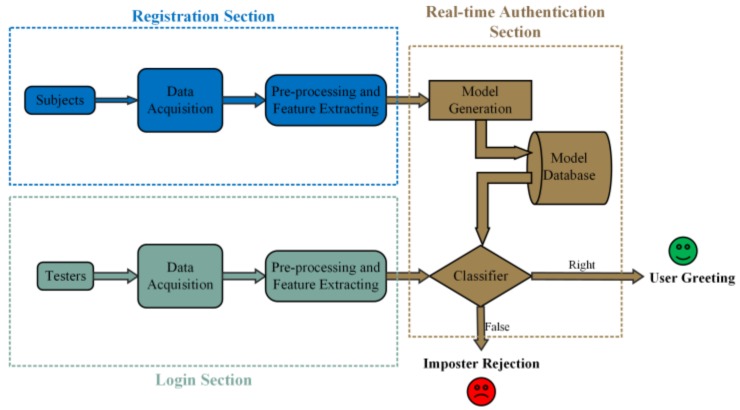
Flowchart of the identity authentication system design.

The overall design of the authentication system is shown in Figure 1. During the registration section, the user and his/her corresponding imposters are asked to focus on the face-RSVP stimulus and make natural eye blinks respectively, and the EEG signals and EOG signals are collected to generate the model of the specific user. The model is stored in the database to provide data support for the classifier in the next phase. In the login section, the same stimulus and instructions are shown to the tester, whose EEG signals and EOG signals are submitted to the classifier for judgment.

#### 2.1.2. Main Framework of the Multi-task Authentication Method

**Figure 2:**
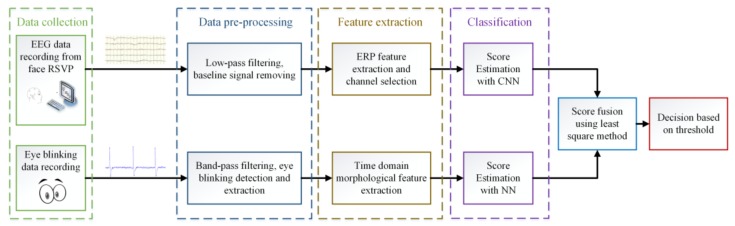
Flowchart of the multi-task authentication method.

As shown in Figure 2, a score fusion module is added after the classification stage for our multi-task authentication system. The detail of each stage will be described below.

### 2.2. Participants

**Figure 3:**
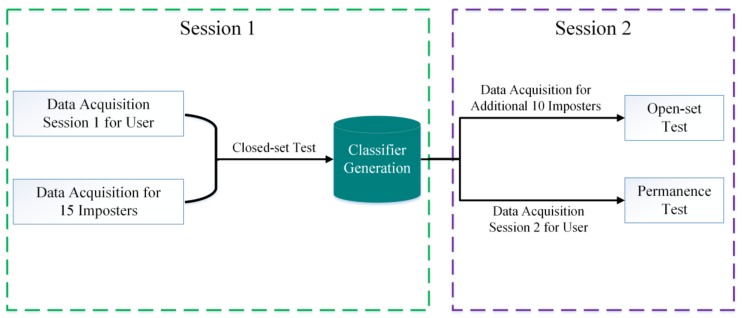
Flowchart of data acquisition and performance test for a user.

We recruited 40 subjects (30 males and 10 females) in the age range of 19 to 23 for the experiment. Among them, 15 are users, 15 are the corresponding imposters, who are used to test the performance in the closed-set (in this paper, it means to authenticate the subjects who have registered in the system). The remaining 10 subjects are imposters, who are used to measurement the performance in the open-set (in this paper, it means to detect the imposters who have not registered in the system). As shown in Figure 3, the data acquisition consists of two sessions. In the session 1, the datasets of each user and his/her corresponding 15 imposters are collected. The closed-set authentication tests are performed based on these datasets, and a classifier is generated for each user simultaneously. In the session 2, 10 additional imposters’ datasets are acquired to estimate the open-set capability of the system. And another data acquisition session is conducted for each user to evaluate the permanence of the system, with an average time interval of 30 days from the first acquisition. Both the open-set test and permanence test are performed on the classifiers generated from the session 1.

All participants are college students, right-handed, and have normal or corrected-to-normal visual ability. None of the participants had a history of neurological disease. This study was conducted after we acquired informed consent and Ethics Committee approval of China National Digital Switching System Engineering and Technological Research Center. All of the participants have signed a written informed consent before participating and obtained a payment after completing the experiment.

### 2.3. Data Acquisition

#### 2.3.1. Visual Evoked EEG Acquisition

##### Self- or Non-Self-Face RSVP Paradigm

**Figure 4:**
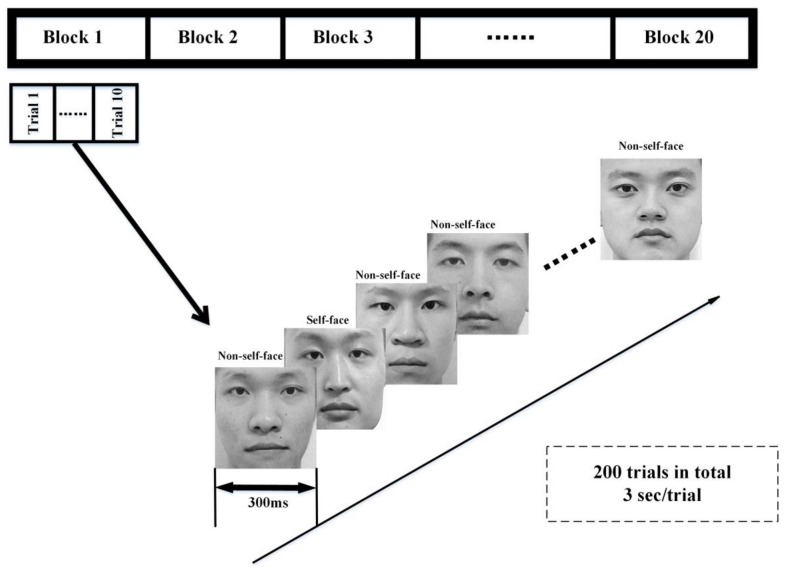
Flowchart of the self- or non-self-face RSVP paradigm.

In our experiment, the RSVP is composed of self- or non-self-face images; the self-face images stand for the user’s own face, and the non-self-face images include both his/her familiar faces or unfamiliar faces. Thus, for each user, there is a specific visual evoked sequence. Each RSVP trial is composed of one self-face image and nine non-self-face images, and the presentation time of each image is 300 ms. The presentation order of the self- or non-self-face images in each trial is randomized to avoid the effect of subject prediction on the next stimulus. The dataset consists of 20 blocks, and each block consists of 10 trials (for the trials in the same block, 10 of the face images are the same but in different random order), as shown in Figure 4. The experiment is conducted in a quiet environment without light interference and electromagnetic interference. A short rest comes after 10 blocks. Each subject has 200 trials in our dataset.

All face images present only facial information and no expression. Each image is resized to 400 × 400 pixels. All the face images are collected from the college student volunteers, and are used for academic research after obtaining their written informed consent. The RSVP stimulus is written in Qt5.5.0 (a cross-platform C++ graphical user interface application development framework developed by Qt Company) and is presented at the center of the screen with a refresh rate of 60 Hz.

##### EEG Data Acquisition

For each user, there is a specific face RSVP to evoke a specific EEG model. During the face RSVP presentation, we ask each user to focus on his own face images and count the number of occurrences of the self-face images in his minds. For the corresponding imposters of a user (assume that user A), they receive the same face RSVP stimulus as user A. Thus, in the session 1 and session 2, there are 225 (15 users, 15 imposter datasets per user) imposter datasets and 150 (15 users, 10 imposter datasets per user) imposter datasets in total, respectively.

**Figure 5:**
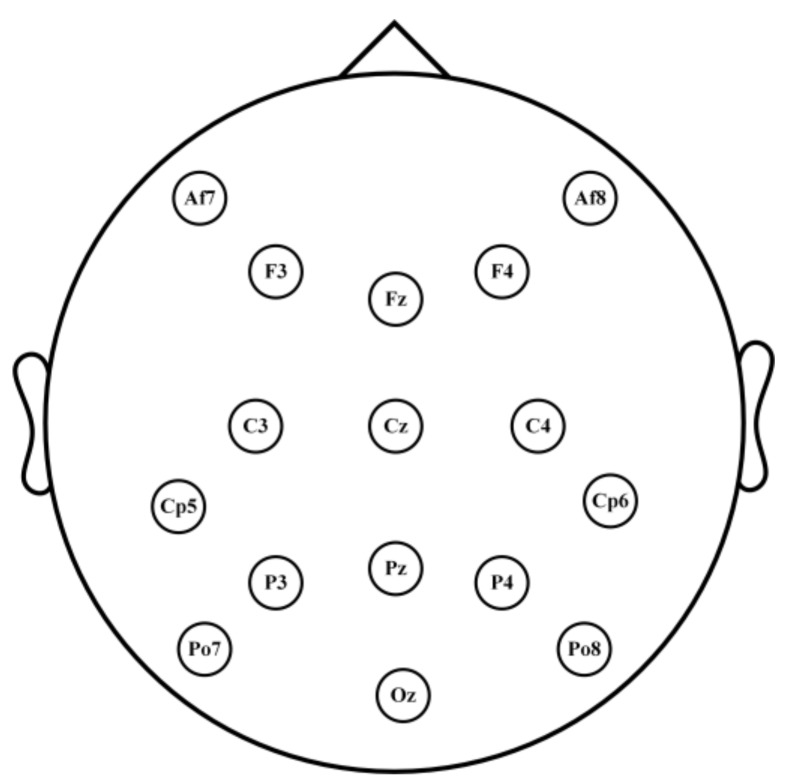
Electrode positions of the 16 channels.

The EEG signals are recorded using a g.USBamp amplifier with 16 wet active electrodes. The sampling rate is 2400 Hz. As shown in Figure 5, the 16 channels are as follows: Fz, Cz, P3, Pz, P4, Po7, Oz, Po8, C3, C4, F3, F4, Af7, Af8, Cp5, and Cp6.

#### 2.3.2. Eye Blinking Data Acquisition

The eye blinking signals are also recorded by the g.USBamp amplifier, but only one electrode is used, namely FP1. The sampling rate is also 2400 Hz. In this stage, each subject was asked to make 10–12 natural eye blinks in each trial with duration of 40 s. A total of 10 trials were recorded. During the recording phase, the subjects were asked to stay calm and not do any body or eye movements as possible. Finally, we randomly pick 100 eye blinking signals for each subject for analysis.

### 2.4. Data Preprocessing

#### 2.4.1. Preprocessing of EEG Signals

The raw EEG data are filtered by a low-pass Chebyshev digital filter with a passband of 40 Hz and a stopband of 49 Hz for further analysis [27]. Then the data are downsampled from 2400 Hz to 600 Hz by averaging four consecutive samples. Finally, the data are epoched to a range of −200 ms to 1000 ms with respect to stimulus onset, and the former interval data from −200 ms to 0 ms are used as the baseline.

#### 2.4.2. Preprocessing of Eye Blinking Signals

**Figure 6:**
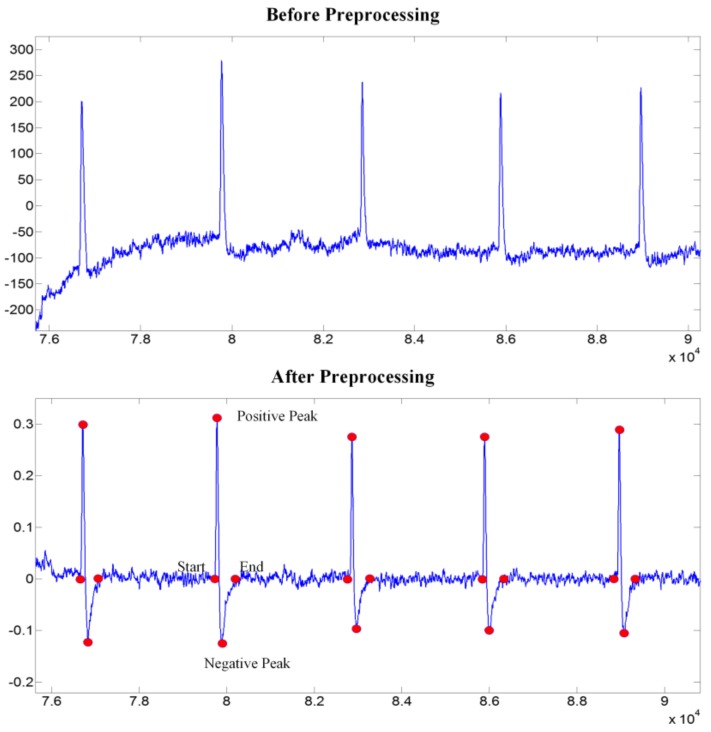
The contrast of before and after preprocessing of the eye blinking signals.

The primary purpose of preprocessing is to extract eye blinking signals from EEG signals, which are acquired from the Fp1 electrode. The main frequency of the EOG signals is concentrated in the 0.1 Hz to 15 Hz. Thus, firstly, the raw data are filtered by a band-pass Chebyshev digital filter with a passband of 1 Hz and 40 Hz. Then the data are downsampled from 2400 Hz to 600 Hz by averaging four consecutive samples. The eye blink can cause a distinct peak in the EEG signal. Therefore, a threshold can be applied to find the positive and negative peaks of the eye blinking signals. It can be concluded from the data we acquired that the positive peaks is always higher than 100 μV and the negative peaks is always smaller than −50 μV. After finding the peaks of eye blinking signals, the sample index is decreased (increased) from the positive peak (negative peak) until the first zero crossing is detected to determine the start (end) of an eye blinking signal. Finally, the eye blinking signals are normalized to −1~1 by dividing the maximum amplitude of the whole eye blinking signal peaks. The contrast of before and after preprocessing of the eye blinking signals is shown in Figure 6.

### 2.5. Feature Extraction

#### 2.5.1. Feature Extraction of EEG

**Figure 7:**
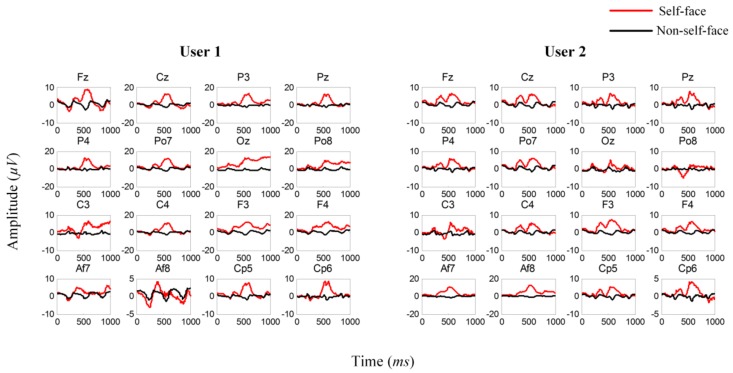
Averaged ERPs of self-face and non-self-face stimuli in two different users.

To gain a comprehensive understanding of our data, we average the ERPs elicited by self-face and non-self-face stimuli. The results show an obvious distinction in the stimuli of different categories, and the latency and amplitude of the ERP components vary in different individuals, as shown in Figure 7.

Therefore, selecting the specific channels for each user is important. Our selection method is based on the algorithm proposed by Yeom et al. [14]. First, we calculate the pointwise biserial correlation coefficient (referred to as the p-value in the following discussion) for each channel. The p-value is a special form of the Pearson product-moment correlation coefficient and is defined as follows:

(1) $$P_{i}(n)=\frac{\sqrt{N_{1}N_{2}}}{N_{1}+N_{2}}\cdot \frac{M_{i}^{SF}(n)-M_{i}^{NSF}(n)}{S(n)}$$

where i denotes the number of channels, namely, i = 1, 2, …, 16; and n represents the sample point, namely, n = 1, 2, …, 600. $N_{1}$ and $N_{2}$ are the total numbers of trials of the self-face and non-self-face stimuli, respectively. $M_{i}^{SF}(n)$ and $M_{i}^{NSF}(n)$ are the mean values of all trials in both classes on the sample point n. S(n) denotes the standard deviation of all trials of both self-face and non-self-face stimuli. $P_{i}(n)$ increases when the EEG signals are further apart when facing the two different stimuli or when the variance is smaller. As a result, the channels and time intervals with a high *p*-value are supposed to be the representative domain for person authentication.

Then, we select 200 sample points whose p-value is ranked in the top 200 in each channel. Next, the mean squares of the selected 200 sample points for each channel are calculated and sorted in a descending order. The channels whose mean square is in the top five are finally selected. Thus, the five channels and the corresponding time intervals with 200 sample points in each trial are chosen for the following analysis.

**Figure 8:**
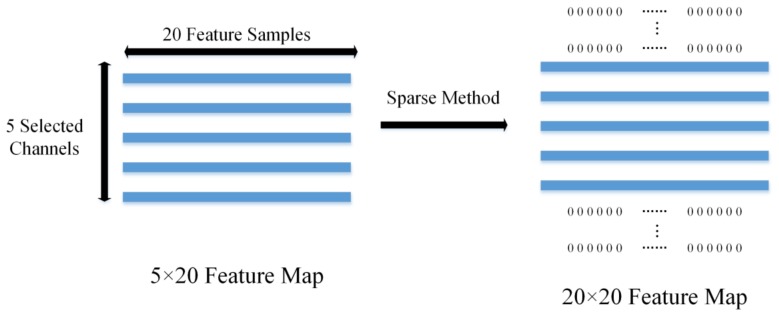
The changes of EEG feature map.

The features are calculated from the data with respect to the self-face images stimulus onset, which only uses the selected channels and time intervals by averaging voltages in 20 non-overlapping time-windows with a width of 10 sample points, so the feature per trial is a 5 × 20 matrix. In order to organize the features to feed CNN for training, we expand the 5 × 20 matrixes to 20 × 20 matrixes using the sparse method, namely adding zero in the blank space, as shown in Figure 8. In this way, the 20 × 20 feature map is generated for each trial.

#### 2.5.2. Feature Extraction of Eye Blinking Signals

**Figure 9:**
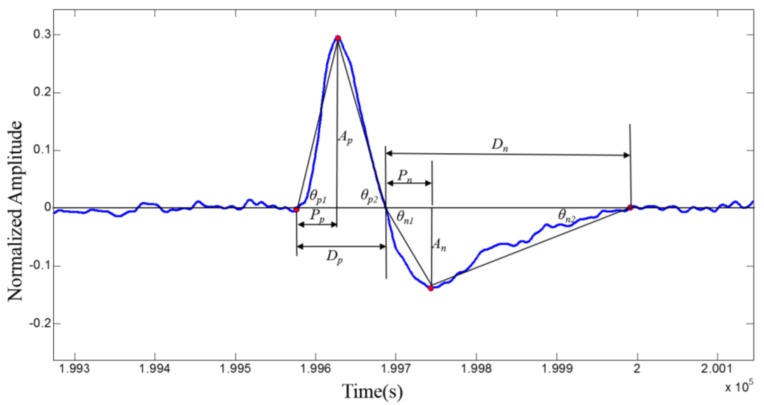
Time domain morphological features of the eye blinking signals.

In this paper, we extract the time domain morphological features of the eye blinking signals. The features can be divided into the following four categories: energy features, position features, slope features, and derivative features. The detail definition of each category is shown in Figure 9 and Table 1. After completing the feature extraction of each category, we connect them together to form a 20-dimensional feature vector.

### 2.6. Score Estimation with CNN and NN

According to the characteristics of EEG and eye blinking signals, we adopt two different classification methods to obtain a better result.

#### 2.6.1. Score Estimation of EEG Features with CNN

**Figure 10:**
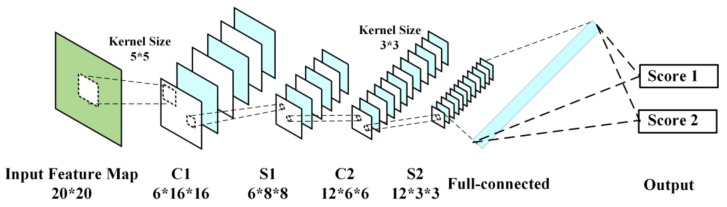
The architecture of the CNN in this paper.

Convolutional neural network (CNN) is a kind of artificial neural network, which has become a hot research topic in the field of speech analysis and image recognition. Its weight-sharing network structure makes it more similar to the biological neural network, which reduces the complexity of the network model and the number of weights [28]. The CNN we are using is a four-layer network, which contains two convolution layers and two pooling layers. The limited number of the layers is mainly because of the small size of input feature map. If the network is too complex, the true information of the input maps may vanish [29]. The structure of the CNN in our work is shown in Figure 10.

As it shows, C1 is the first convolution layer with six feature maps, whose kernel size is 5. S1 is the first maxpooling layer with the scale size of 2. C2 is the second convolution layer with 12 feature maps, whose kernel size is 3. S2 is the second maxpooling layer with the same scale size as S1. Finally, there is a full-connected layer with 108 feature points determining the score estimation of input feature map. In the training stage, the learning rate, batch size, and learning epoch are set to 1, 100, and 500, respectively.

#### 2.6.2. Score Estimation of Eye Blinking Features with NN

**Figure 11:**
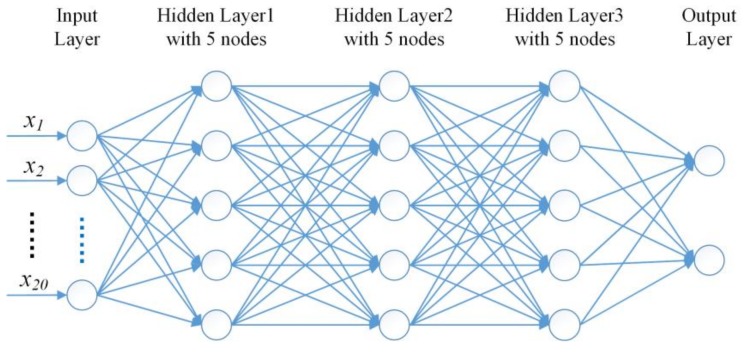
The architecture of the NN in this paper.

For eye blinking feature, we make the score estimation via a back propagation neural network (NN). The NN we design has three hidden layers, and for each hidden layer, there are five neuron nodes. The input layer has 20 nodes corresponding to the eye blinking features. The output layer has two nodes to gain the estimation score of each category. The architecture of the neural network is shown in Figure 11.

### 2.7. Score Fusion Using Least Square Method

In score fusion stage, we propose a least square method based on power series fusion function. The fusion function is defined as follows:

(2) $$S_{f}=f(\vec{\lambda },\vec{s})=\lambda_{0}+\sum_{m=1}^{M}\sum_{n=1}^{N}\lambda_{m,n}S_{n}^{m}$$

where $S_{f}$ is the fusion score, M is the order of power series, N is the number of modality in a fusion system, and $\vec{\lambda }$ is the fusion parameter. It can be changed to the matrix form:

(3) $$S_{f}=f(\vec{\lambda },\vec{s})=g(\vec{s})\cdot \vec{\lambda }$$

namely:

(4) $$g(\vec{s})=[1,s_{1},s_{2},\ldots ,s_{N},\ldots ,s_{1}^{M},s_{2}^{M},\ldots ,s_{N}^{M}]$$

(5) $$\vec{\lambda }={\left[\lambda_{0},\lambda_{1,1},\lambda_{1,2},\ldots ,\lambda_{1,N},\ldots ,\lambda_{M,1},\lambda_{M,2},\ldots ,\lambda_{M,N}\right]}^{T}$$

The method of solving the fusion parameters is discussed below. For a fusion score predicted value $y_{i}$, it can be defined as follows:

(6) $$y_{i}=\left\{ \begin{array}{ll} 1\; & i\in w_{1}\\ 0\; & i\in w_{0} \end{array}\right.\;,\;\;i=1,2,\ldots ,k$$

where i represents a sample with a known category, $w_{1}$ represents the user class, $w_{0}$ represents the imposter class, and k represents the number of samples. The least squares method is used to estimate the estimated values of the fusion parameters, which makes the objective function value minimum, namely:

(7) $$\psi (\vec{\lambda })=\frac{1}{2}{\left\|\vec{y}-g\cdot \vec{\lambda }\right\|}^{2}$$

where:

(8) $$g=[1_{k\times 1}\;g_{1}\;\ldots \;g_{MN}]=\left[\begin{array}{llll} 1 & g_{1}(\vec{s_{1}}) & \ldots & g_{MN}(\vec{s_{1}})\\ 1 & g_{1}(\vec{s_{2}}) & \ldots & g_{MN}(\vec{s_{2}})\\ \ldots & \ldots & \ldots & \ldots \\ 1 & g_{1}(\vec{s_{N}}) & \ldots & g_{MN}(\vec{s_{N}}) \end{array}\right]$$

Finally, the estimated value of fusion parameters can be obtained by the following equation, namely:

(9) $$\tilde{\vec{\lambda }}={\left(g^{T}g\right)}^{-1}g^{T}\vec{y}$$

## 3. Results

### 3.1. Average ERPs Analysis

**Figure 12:**
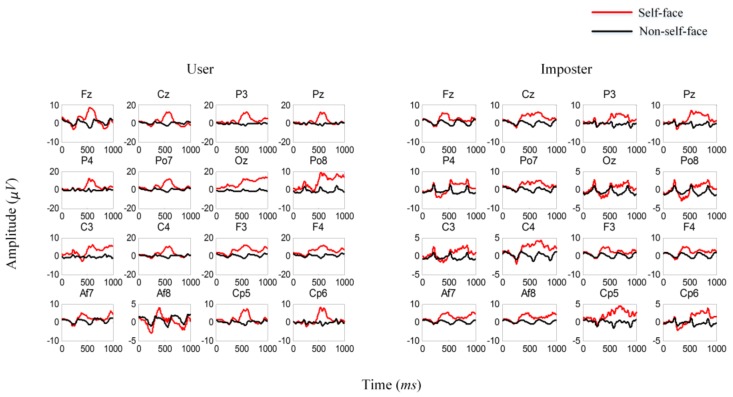
Average ERPs evoked by the self-face (red line) and non-self-face (black line) images.

To validate the effectiveness of self- or non-self-face RSVP paradigm, we analyze the average ERPs in the first stage. The average ERPs of a real user and one of his imposters are shown in Figure 12.

N250, which is a main ERP component related to face stimulus according to previous EEG evidence, can be observed clearly in both user and imposters. For the user, an obvious difference is observed between the ERPs evoked by the self-face and non-self-face images, and the difference is specific to an individual. For the imposter, although a certain difference is observed between the two kinds of ERPs, the amplitude, shape, and latency are distinctly different from those for the user. Furthermore, the channel location of the difference in the imposter varies from that in the user, which justifies channel selection.

**Figure 13:**
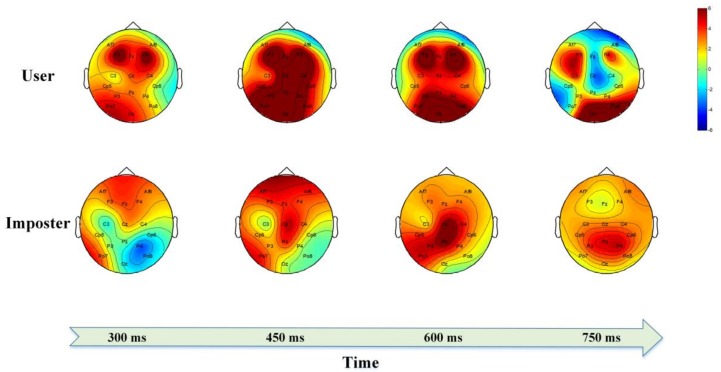
The ERP topographical maps.

The individual differences in the ERP topographical maps of the user and imposter are clearly observable in Figure 13. The brain activation intensity and region is distinctly different between the user and imposter. In summary, individual-specific ERP characteristics are evoked by the self- or non-self-face RSVP paradigms and are difficult to be forged by the imposter.

### 3.2. Closed-Set Authentication Result 

In this stage, we first average two adjacent single EEG trials to obtain more stable and less noisy EEG signals. Therefore, there are 100 average trials EEG signals and 100 eye blinking signals for each user and imposter. Please note that, for one user, there are 15 corresponding imposters. Thus, during the classifier training, there are 100 user samples and 1500 imposter samples, which causes the imbalance of training samples. To solve this problem, we adopt the synthetic minority over-sampling technique (SMOTE). Its basic idea is that synthesizing the new samples according to the minority class samples. The specific algorithm flow is as follows:

(1) For each sample *x* in a few classes, calculate its Euclidean distance to all samples in the minority class sample set, and then obtain its *K* nearest neighbors.
(2) Set a sampling magnification *N* based on the sample imbalance ratio. For each minority sample *x*, randomly select several samples from its *k* neighbors, assuming that the selected neighborhood is $\tilde{x}$.
(3) For each randomly selected neighbor $\tilde{x}$, construct a new sample with the original sample according to the following formula, namely:
  (10) $$x_{new}=x+rand(0,1)\times (x-\tilde{x})$$

Thus, we expand the quantity of user dataset samples (both EEG signals and eye blinking signals) from 100 to 1500 through SMOTE.

In the training and test stage, 1350 out of 1500 feature vectors are selected randomly for classifier generation, and the remaining 150 feature vectors are used to test the classifier.

The classification accuracy (*ACC*), false acceptance rate (*FAR*), and false rejection rate (*FRR*) are used to evaluate the performance of the system of each user, which are defined as follows:

(11) $$ACC=\frac{number\;of\;correctly\;authenticated\;samples}{total\;number\;of\;test\;samlpes}$$

(12) $$FAR=\frac{number\;of\;falsely\;accepted\;samples}{total\;number\;of\;imposter\;test\;samlpes}$$

(13) $$FRR=\frac{number\;of\;falsely\;rejected\;samples}{total\;number\;of\;user\;test\;samlpes}$$

**Figure 14:**
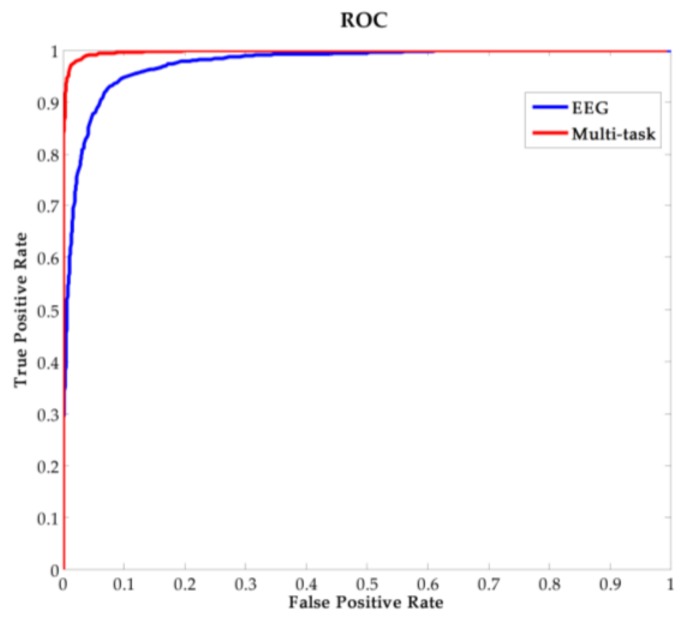
The average ROC curves of EEG-based and multi-task authentication system.

The classification result for the 15 users is shown in Table 2. We can get a mean *ACC* of 92.40%, *FAR* of 6.71%, and *FRR* of 8.49% using EEG data only, which illustrates the feasibility of the face RSVP paradigm we have designed. What inspires us is that the system performance has a significant improvement after combining the eye blinking signal features. It gains a mean *ACC* of 97.60%, *FAR* of 2.71%, and *FRR* of 2.09%, which demonstrates the precision of multi-task authentication system. The average receiver operating characteristic (ROC) curves of EEG-based system and multi-task system are shown in Figure 14.

A distinct improvement of the performance can be concluded from the ROC curves, illustrating the effectiveness of our method. Moreover, for EEG-based authentication system, the time-cost of one time authentication is 6 s (two average EEG trials), and the time to complete a blink would not exceed one second. Thus, for multi-task person authentication system, the time-cost of one time authentication is about 7 s. This time-cost illustrates the real-time capability of our system.

### 3.3. Open-Set Authentication Result

In order to test the security of our system, 10 additional imposters were recruited to invade the system of each user. One hundred trial EEG signals and eye blinking signals of each imposter were acquired. Thus, for each user, there are 1000 imposter samples. These imposter samples are estimated by the classifiers generated from the closed-set.

As shown in Table 3, *FAR* is applied to evaluate the performance of the system in open-set. Using EEG data only, a mean *FAR* of 5.75% is obtained, which illustrates the system is difficult for imposters to cheat. And the mean *FAR* can be decreased to 3.9% when combines eye blinking signals, which proves a better performance of the multi-task authentication system. Therefore, we can conclude from the result that, although in open-set, our system has the ability to identify imposters and reject them.

### 3.4. Permanence Tests for Users

In order to test the system permanence, a second data acquisition session was conducted for each user. The average time interval between the first session and second session is about 30 days. In the second session, 50 trials EEG signals and eye blinking signals of each user were acquired. Thus, there are 50 login tests for each user. These user login tests are estimated by the classifiers generated from the closed-set.

As shown in Table 4, *FRR* is applied to evaluate the permanence tests for each user. Using EEG data only, a mean *FRR* of 7.07% is obtained, and it can be decreased to 3.87% when combines eye blinking signals. The test results illustrate our system has the capability of permanence, which is essential for a person authentication system.

## 4. Discussion

The experimental results have revealed the suitability of the proposed multi-task person authentication system. The multi-task authentication system has a better performance in many aspects. Below, we provide a detailed discussion of our results for a more complete exploration of the EEG-based person authentication systems.

### 4.1. Comparison with the Existing EEG-Based Authentication Systems

An increasing number of studies have been conducted to improve the performance of EEG-based or EEG-based multi-task person authentication systems. A comparison of our method with previous related works is provided in Table 5. The superiority of our proposed method can be seen from the performance comparison.

In terms of accuracy, we obtain a better result compared to most of the previous works. For example, Patel et al. [23] achieved a mean *ACC* of 92.5% in 10 users. In our method, with a larger database, the mean *ACC* of 97.6% is higher. Although our *ACC* is a little lower than Abo-Zahhad et al. [25], the quantity of our data samples is much bigger (we have 1500 data samples for each person, but there are only 50 data samples for each person in their database). Thus, we can get a more robust result.

In terms of time cost, we perform a satisfactory real-time capability. In previous works, it always takes over 10 s to complete one-time authentication. In this paper, the time cost has been decreased to 7 s with a high authentication accuracy.

Another essential work in this paper is the open-set authentication tests for additional imposters, which is not employed in previous EEG-based person authentication systems. For a practical authentication system, a satisfying performance in closed-set is not enough. A low *FAR* in open-set authentication test can ensure systems would not be cheated by the outsiders. In this paper, with 1000 intrusion tests for each user, a mean *FAR* of 3.9% is accomplished, which illustrates the robust security of our systems. Moreover, permanence tests for user are conducted in this work, which prove the reliability of our system.

### 4.2. Future Research Directions 

Our method reveals the potential of using EEG and eye blinking signals as an ideal biometric. However, there are something we need to consider in the future work.

On the one hand, most data acquisition of EEG is inconvenient at the current stage of the art. We have to place many electrodes on the scalp and use conductive gel to reduce skin impedance. Thus, channel selection is adopted in this paper, which can not only improve the accuracy of the system, but also ameliorate the portability. Moreover, with the development of technology, wireless EEG devices with dry electrodes have been produced. Although the signal quality of these devices is poor, this is the first step for practical application.

On the other hand, the permanence of EEG signals needs further exploration. EEG signals may change with age, which would influence the performance of system. Therefore, it’s meaningful and necessary to repeat the experiment after a few months or even a few years to explore the permanence of EEG-based biometric. Furthermore, major EEG experiment is conducted in the normal state of subjects or in a quiet environment without noise. In practical life, the external factors, such as fatigue, mood, heart rate, alcohol, drugs and noisy surrounding environment, should be considered and tested.

## 5. Conclusions

In this paper, inspired by the excellent performance of EEG signals in forgery prevention, we design a multi-task EEG-based person authentication system combining eye blinking signals. A distinct and stable EEG-biometric is elicited from the self- or non-self-face RSVP paradigm with a low time cost of 6 s. Eye blinking signals, which are easy to obtain and have a high discrimination over different subjects, are employed to enhance the robustness and accuracy of EEG-based authentication system. Satisfactory experiment results with high accuracy and real-time capability have been achieved. Moreover, the robust performance of open-set authentication tests and permanence tests demonstrates the high security of our systems. In future work, we will repeat the experiment after a few months to explore system stability. Commercial portable EEG acquisition equipment, such as the Emotiv EPOC headset, will be used to improve system practicability. Moreover, other EEG artifacts, such as electro-myogram (EMG) from teeth biting and EOG from eye movement, may be adopted to enhance the system performance.

## Figures and Tables

**Table 1:** The detail definition of each feature.

Category	Symbol	Definition
Energy features	*A_p_*	Amplitude of positive peak of the eye blinking signal
	*A_n_*	Amplitude of negative peak of the eye blinking signal
	*E_p_*	Area under positive pulse of the eye blinking signal
	*E_n_*	Area under negative pulse of the eye blinking signal
Position features	*P_p_*	Position of positive peak of the eye blinking signal
	*P_n_*	Position of negative peak of the eye blinking signal
	*D_p_*	Duration of positive pulse of the eye blinking signal
	*D_n_*	Duration of negative pulse of the eye blinking signal
Slope features	*S_1_*	Slope of the onset of the positive pulse (tan(*θ_p_*_1_))
	*S_2_*	Slope of the onset of the negative pulse (tan(*θ_n_*_1_))
	*S_3_*	Slope of the offset of the positive pulse (tan(*θ_p_*_2_))
	*S_4_*	Slope of the offset of the negative pulse (tan(*θ_n_*_2_))
	*S_5_*	Dispersion degree of the positive pulse (σ_p_/mean_p_)
	*S_6_*	Dispersion degree of the negative pulse (σ_n_ /mean_n_)
Derivative features	*D_1_*	Amplitude of positive peak of first derivative
	*D_2_*	Amplitude of negative peak of first derivative
	*D_3_*	Position of the positive peak of first derivative
	*D_4_*	Position of the negative peak of first derivative
	*D_5_*	Number of zero crossings of the first derivative
	*D_6_*	Number of zero crossings of the second derivative

**Table 2:** Performance of EEG-based and multi-task person authentication system in closed-set.

	*ACC* (%)		*FAR* (%)		*FRR* (%)	
Users	EEG	Multi-Task	EEG	Multi-Task	EEG	Multi-Task
1	91.33	99.67	6.00	0.67	11.33	0.00
2	96.67	98.00	3.33	1.33	3.33	2.67
3	98.00	98.00	3.33	3.33	0.67	0.67
4	93.67	98.33	6.00	2.00	6.67	1.33
5	88.33	95.67	8.00	4.67	15.33	4.00
6	97.67	98.33	2.00	2.00	2.67	1.33
7	85.00	96.00	14.67	3.33	15.33	4.67
8	90.67	99.67	4.67	0.67	14.00	0.00
9	90.67	94.67	8.00	6.67	10.67	4.00
10	94.33	97.67	7.33	2.67	4.00	2.00
11	93.33	95.00	6.00	6.00	7.33	4.00
12	93.33	98.00	6.67	2.67	6.67	1.33
13	91.67	96.67	7.33	3.33	9.33	3.33
14	91.67	99.67	9.33	0.00	7.33	0.67
15	89.67	98.67	8.00	1.33	12.67	1.33
Mean (std)	92.40 (3.50)	97.60 (1.65)	**6.71** **(3.01)**	**2.71** **(1.93)**	**8.49** **(4.68)**	**2.09** **(1.57)**

**Table 3:** The performance of EEG-based and multi-task authentication system in open-set.

*FAR* (%)		
User	EEG	Multi-Task
1	3.60	2.70
2	3.90	3.00
3	1.30	1.40
4	6.30	5.50
5	5.70	4.70
6	2.50	1.30
7	12.90	5.80
8	5.10	2.20
9	8.70	6.30
10	6.70	3.90
11	3.80	4.00
12	4.00	1.90
13	4.50	8.90
14	3.40	2.10
15	13.80	4.80
Mean (std)	5.75 (3.57)	3.90 (2.13)

**Table 4:** Permanence test results of each user.

*FRR* (%)		
User	EEG	Multi-Task
1	6.00	2.00
2	0.00	0.00
3	2.00	0.00
4	12.00	16.00
5	16.00	8.00
6	2.00	6.00
7	10.00	6.00
8	14.00	0.00
9	8.00	6.00
10	4.00	4.00
11	2.00	2.00
12	2.00	4.00
13	10.00	2.00
14	10.00	0.00
15	8.00	2.00
Mean (std)	7.07 (4.95)	3.87 (4.24)

**Table 5:** Performance comparison of the previous works.

Author	Data Type	Time Cost (s)	*ACC* (%)	*FAR* (%)	*FRR* (%)	Open-Set Test	Permanence Test
**Armstrong et al. [30]**	Text reading related EEG	Not mentioned	89	Not mentioned	Not mentioned	None	Yes
**Yeom et al. [14]**	Visual evoked related EEG	31.5~41	86.1	13.9	13.9	None	None
**Marcel et al. [31]**	Motor imagery related EEG	15	80.7	14.4	24.3	None	None
**Zhendong et al. [32]**	Visual evoked related EEG	6.5	87.3	5.5	5.6	None	None
**Patel et al. [23]**	EEG and Hand synergies	8	92.5	Not mentioned	Not mentioned	None	None
**Abo-Zahhad et al. [25]**	EEG and eye blinking signal	Not mentioned	98.65	Not mentioned	Not mentioned	None	None
**This paper**	EEG and eye blinking signal	7	97.6	2.71	2.09	Yes	Yes
